# Phylogeography and ecological niche modeling suggest southward expansion of *Morinda officinalis* How in China

**DOI:** 10.3389/fpls.2025.1643733

**Published:** 2025-11-21

**Authors:** Chaoyu Zhang, Miao Zhang, Xuehui Huang, Qingtong Lan, Hua Zhu, Yong Huang, Hui Tian

**Affiliations:** 1 Faculty of Pharmacy, Guangxi University of Chinese Medicine, Nanning, Guangxi Zhuang Autonomous Region, China; 2 Guangxi Key Laboratory of Zhuang and Yao Ethnic Medicine, Zhuang and Yao Medicine Collaborative Innovation Center, Nanning, Guangxi Zhuang Autonomous Region, China; 3 University Engineering Research Center of Characteristic Traditional Chinese Medicine and Ethnomedicine, Guangxi, Nanning, Guangxi Zhuang Autonomous Region, China

**Keywords:** phylogeography, ecological niche modeling, *Morinda officinalis*, evolutionary history, divergence time, historical dynamics

## Abstract

*Morinda officinalis* How is a traditional medicine plant that is currently native to the tropical and subtropical mainland as well as the islands of China. The effects of geological movements and Quaternary climate fluctuations on *M. officinalis* may be analyzed by genealogical geography in conjunction with ecological niche simulation. We performed Bayesian phylogenetic analysis of *M. officinalis* using chloroplast (*rbcL*, *matK*, and *trnH-psbA*) and nuclear gene sequence (*ITS2*) genealogy and simulation of the distribution of *M. officinalis* in the Chinese region. Low nucleotide and haplotype diversity, and genetic geographic structure of *M. officinalis* indicated the separation of the species into two lineages at 35.91 Mya. The optimal habitat of *M. officinalis* varied greatly during the Last Interglacial, Last Holocene, and Middle Holocene periods as well as in the current period. The species experienced expansion during the Last Interglacial and contraction during the Last Holocene. A large-scale migration occurred from the Tibetan Plateau to southeastern China, and the Shiwan and Liuwan Mountains in southern Guangxi, as well as the Dinghu Mountains in Zhaoqing, Guangdong, and the Ehuangzhang Mountains in Yangjiang, Guangdong, which were the principal Quaternary ice age refuges for *M. officinalis*. The island lineages of *M. officinalis* diverged before the emergence of the Qiongzhou Strait. The historical ancestral origin of *M. officinalis* is thought to be the Tibetan Plateau and the southward migration in the early Miocene and subsequent *in situ* diversification may explain the diversity of *M. officinalis*. Our results provide phylogenetic evidence for the origin of *M. officinalis*, reveal the process of diversification, and indicate that the species adapted to a timeline of major geological and climatic episodes rather than localized, episodic, and rate-varying events.

## Introduction

The Quaternary glacial-interglacial cycles (2.58 Mya-present) profoundly shaped global biodiversity patterns and drove repeated range contractions, expansions, and genetic diversification across taxa (Alvarado-Serrano and Knowles, 2020). Ice sheets and arid conditions displaced temperate and subtropical species into fragmented refugia during the Last Glacial Maximum (LGM, ~21 ka), with postglacial recolonization routes imprinting lasting signatures on contemporary genetic and ecological landscapes (Guo et al., 2024). A global biodiversity hotspot, complex topography, and monsoon dynamics buffered species extinction risks in east Asia, and fostered intricate networks of microrefugia and migration corridors that defy simplistic latitudinal shift models (Yin et al., 2015). The unique interplay of paleogeography, climate heterogeneity, and biotic interactions made east Asia as a natural laboratory for testing biogeographic theories, particularly how subtropical evergreens, unlike their temperate counterparts, navigated Quaternary climatic upheaval (Qiu et al., 2017).

Phylogeography and ecological niche modeling (ENM) have emerged as synergistic tools to disentangle the dynamics of species range, and diversification. Phylogeography reconstructs historical dispersal and demographic processes, whereas ENM quantifies climatic suitability across time and links microevolutionary patterns, e.g., lineage divergence, to macroecological processes, e.g., niche conservatism (Alvarado-Serrano and Knowles, 2014). For example, integrated studies revealed that glacial cooling compressed the ranges of *Quercus kerrii* southward, with postglacial warming triggering northward expansion and genetic bottlenecks in recolonized zones, which is a pattern that now is recognized as a template for temperate taxa (Jiang et al., 2018). However, subtropical evergreens, particularly understory plants with limited dispersal and edaphic specialization, may have responded differently by prioritizing *in situ* persistence over rapid migration. This divergence challenges the universality of temperate-centric biogeographic models and underscores the need for clade-specific investigations.

Paleo-land bridges, e.g., exposed continental shelves during glacial lowstands, and island refugia, e.g., Hainan and Taiwan, likely mediated bidirectional species exchanges, although the roles of these bridges remain contested (Rui et al., 2024). Whereas fossil pollen and genetic data suggest southward shifts of subtropical forests into unglaciated refugia during the LGM (Song et al., 2024), ENM for certain species indicate paradoxical range expansions into warmer southern niches. These contradictions highlight unresolved questions: Did subtropical evergreens track shifting climates or persist in microrefugia? How did dispersal barriers, e.g., straits and mountains, and biotic interactions, e.g., frugivore dependencies, modulate the responses of these species?

The East Asian-Southeast Asian biogeographic corridor is a dynamic nexus of continental and insular ecosystems, which has long served as both a glacial refuge and a postglacial expansion pathway for flora (Woodruff, 2010). This region’s unique geoclimatic features, including latitudinal alignment of mountain ranges, e.g., Nanling and Wuyi Mountains, paleo-land bridges that connect mainland China to Hainan and Taiwan islands, and monsoon-driven humidity gradients, created a mosaic of microrefugia during the Quaternary period (Tian et al., 2018). These heterogeneous habitats allowed species to persist *in situ* or migrate along elevational and coastal-inland axes, which contrasts with the simpler latitudinal shifts observed in Europe and North America (López-Pujol et al., 2011). For instance, fossil records from karst caves in southern China indicated that subtropical broadleaved forests thrived in localized humid pockets during the LGM (Wang et al., 2015), whereas phylogeographic analyses of T*aiwania cryptomerioides* revealed deep divergence between island and mainland populations, which suggested prolonged isolation (Chou et al., 2011). However, the extent to which these insular systems functioned as independent refugia or stepping stones for bidirectional dispersal remains contentious (Yamada et al., 2021).

The "southward contraction–northward expansion" hypothesis—which proposes that subtropical species retreated to low-latitude refugia during glaciation and later recolonized northern regions —has been a central paradigm in phylogeography (Stewart et al., 2010). This model aligns with genetic patterns in temperate taxa such as *Ginkgo biloba* (Gong et al., 2008), however, the applicability of the model to subtropical evergreens is increasingly challenged. For example, ENM for *Cyclobalanopsis glauca* suggests that suitable habitats expanded southward during the LGM due to increased aridity in northern regions, which contradicts the classical northward retreat narrative (Zhang et al., 2022). Similarly, phylogeographic analyses of *Machilus thunbergii* revealed cryptic refugia along the East China Sea continental shelf, which implies that sea-level fluctuations, not just temperature, dictated range dynamics (Jiang et al., 2024). These discrepancies underscore the need to reevaluate species response to climate and environmental change in light of species-specific ecological tolerances and regional paleogeographic complexities (Lawing, 2021).

Most studies of ecosystem expansion focus on temperate trees or alpine herbs, but neglect economically vital subtropical medicinal plants whose biogeographic histories are further complicated by anthropogenic pressures, e.g., overharvesting and habitat fragmentation (Groner et al., 2022). *Morinda officinalis* How (Rubiaceae) is a karst-adapted medicinal vine with a disjunct East Asian distribution that epitomizes these challenges. *M. officinalis*, a perennial evergreen plant endemic to subtropical East Asia, exemplifies the complex interplay of biogeographic history and anthropogenic pressures that shape medicinal plant distribution. Renowned for its anti-inflammatory and neuroprotective properties, dried root of *M. officinalis* is a well-known traditional Chinese medicine that treats erectile dysfunction, nocturnal emissions, uterine cold infertility, rheumatism, and soft muscle and bone fistulas, with escalating commercial demand driving overharvesting and habitat fragmentation (Li et al., 2024). Current populations are scattered across mainland China, e.g., Guangdong Province and Guangxi Zhuang Autonomous Region, Hainan Island, and northern Vietnam, which may reflect Quaternary climate-driven fragmentation, human-mediated dispersal, or both. However, this ambiguity impedes evidence-based conservation. *M. officinalis* often is restricted to karst limestone habitats, which is a niche preference linked to calcium-rich soils and shaded understories. This fragmented distribution raises critical questions: Is the range of *M. officinalis* a legacy of Quaternary climate-driven contractions or does this distribution reflect recent anthropogenic dispersal? Are island populations of the species evolutionarily distinct units that warrant prioritized conservation?

This study integrates multi-locus genetic analyses and ENM across temporal scales to resolve the phylogeographic ambiguities and climatic drivers that shape the distribution of *M. officinalis*. We employ a dual approach that aims to assess lineage divergence and gene flow by multi-gene phylogeography using chloroplast sequences (cpDNA:*rbcL*, *matK*, and *trnH-psbA*) and nuclear gene sequences (nrDNA:*ITS2*), and to test hypotheses of southward expansion by climatic niche reconstructions for the LGM, mid-Holocene, and present periods.

## Materials and methods

### Plant materials

**Figure 1:**
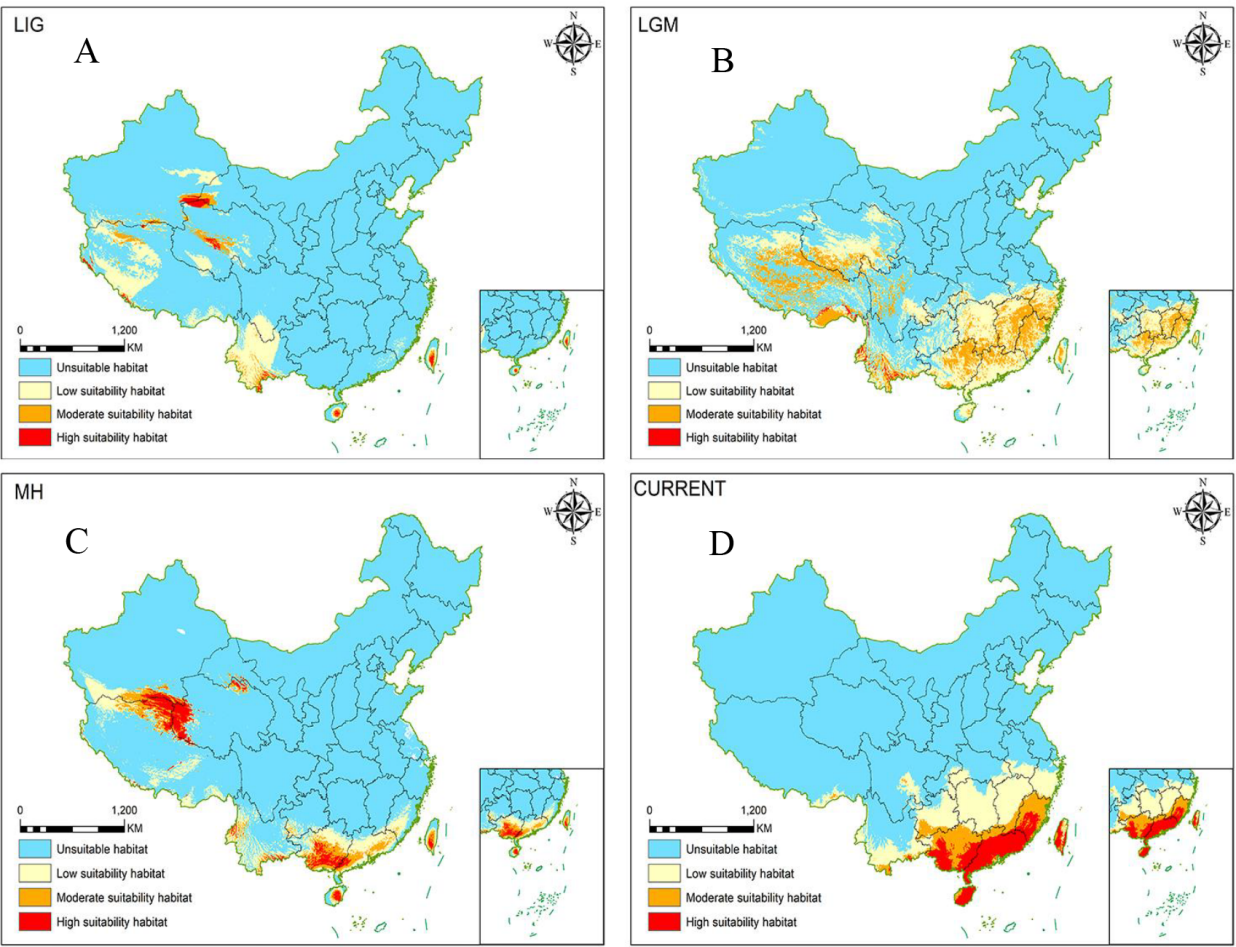
MaxEnt simulation of potential range changes of *Morinda officinalis* over time [Review No. GS(2019)1822]. **(A)** LIG; **(B)** LGM; **(C)** MH, **(D)** Modern (current).

Samples were collected from 286 individuals of *M. officinalis* from 33 localities in China (**Table 1**). The samples covered almost all known populations of the species in the country. Despite the reported potential distribution of *M. officinalis* in Taiwan as indicated by our ecological niche model (**Figure 1D**), samples from this region were not included in the genetic analysis. This was primarily due to challenges in obtaining collection permits from the relevant authorities during the study period, coupled with the reported scarcity and inaccessibility of wild populations, which are often located in remote or protected areas. All *M. officinalis* specimens were deposited in the Herbarium of Guangxi Key Laboratory of Zhuang and Yao Ethnic Medicine (**Table 1**).

**Table 1:** Sample localities, sample sizes, number of haplotypes (N), haplotype diversity (h), and nucleotide diversity (π) for *Morinda officinalis*.

NO.	Abbreviated name of sample Locality	Sample Locality	Sample size	Longitude	Latitude	Genetic diversity		
						N	h	π
1	AP	Anping Town, Cenxi City, Guangxi Zhuang Autonomous Region, China	7	111.064	23.163	1	0.00000	0.00000
2	BBQ	Bapu District, Hezhou, Guangxi Zhuang Autonomous Region, China	10	111.622	23.999	1	0.00000	0.00000
3	BL	Boro County, Huizhou City, Guangdong Province, China	5	114.176	23.239	1	0.00000	0.00000
4	BT	Baoting County, Hainan Province, China	10	109.583	18.718	2	0.20000 ± 0.154	0.00009 ± 0.00007
5	BTZ	Botang Town, Cenxi City, Guangxi Zhuang Autonomous Region, China	10	110.841	22.981	1	0.00000	0.00000
6	DL	Dacheng Town, Fangcheng District, Guangxi Zhuang Autonomous Region, China	8	108.135	21.87	7	0.96429 ± 0.077	0.00151 ± 0.00034
7	DQ	Deqing County, Zhaoqing City, Guangdong Province, China	10	111.938	23.318	1	0.00000	0.00000
8	DX	Dongxing City, Guangxi Zhuang Autonomous Region, China	8	107.995	21.607	6	0.92857 ± 0.084	0.00099 ± 0.00022
9	DZ	Danzhou City, Hainan Province, China	10	109.283	19.896	2	0.46667 ± 0.132	0.00022 ± 0.00006
10	GN	Guangning County, Zhaoqing City, Guangdong Province, China	9	112.387	23.537	2	0.55600 ± 0.090	0.00026 ± 0.00004
11	GY	Gaoyao District, Zhaoqing City, Guangdong Province, China	6	112.287	23.171	2	0.33333 ± 0.215	0.00016 ± 0.00010
12	HA	Hua’an County, Zhangzhou City, Fujian Province, China	10	117.636	25.006	1	0.00000	0.00000
13	HX	Hexi Township, Jingnan County, Zhangzhou City, Fujian Province, China	10	117.291	24.909	1	0.00000	0.00000
14	LD	Ledong City, Hainan Province, China	10	108.755	18.502	2	0.20000 ± 0.154	0.00009 ± 0.00007
15	LM	Longmen County, Huizhou City, Guangdong Province, China	10	114.313	23.597	1	0.00000	0.00000
16	LS	Longshan Township, Jingnan County, Zhangzhou City, Fujian Province, China	10	117.413	24.713	1	0.00000	0.00000
17	LW	Longwen District, Zhangzhou City, Fujian Province, China	7	117.747	24.567	1	0.00000	0.00000
18	LY	Longyan City, Fujian Province, China	10	116.886	24.476	1	0.00000	0.00000
19	MM	Maoming, Guangdong Province, China	7	111.385	21.842	2	0.47619 ± 0.171	0.00022 ± 0.00008
20	NL	Naliang Town, Fangchenggang City, Guangxi Zhuang Autonomous Region, China	10	107.78	21.75	4	0.73333 ± 0.120	0.00048 ± 0.00012
21	NN	Nanning City, Guangxi Zhuang Autonomous Region, China	8	108.383	22.856	3	0.46429 ± 0.200	0.00044 ± 0.00023
22	PB	Pubei County, Qinzhou City, Guangxi Zhuang Autonomous Region, China	10	109.531	22.079	3	0.37778 ± 0.181	0.00052 ± 0.00024
23	PH	Pinghe County, Zhangzhou City, Fujian Province, China	9	117.167	24.132	2	0.22222 ± 0.166	0.00010 ± 0.00008
24	PN	Puning City, Jieyang City, Guangdong Province, China	6	115.806	23.192	1	0.00000	0.00000
25	QZ	Qiongzhong Li and Miao autonomous county, Hainan province, China	10	109.657	19.086	1	0.00000	0.00000
26	RX	Rong County, Yulin City, Guangxi Zhuang Autonomous Region, China	8	110.335	22.958	1	0.00000	0.00000
27	SH	Sihui City, Guangdong Province, China	10	112.528	23.335	1	0.00000	0.00000
28	SS	Shangsi County, Fangchenggang City, Guangxi Zhuang Autonomous Region, China	6	107.63	21.785	1	0.00000	0.00000
29	TX	Fuji County, Wuzhou City, Guangxi Zhuang Autonomous Region, China	9	110.674	23.711	1	0.00000	0.00000
30	WZS	Wuzhishan City, Hainan Province, China	9	109.688	18.914	2	0.22222 ± 0.166	0.00010 ± 0.00008
31	YJ	Yangjiang City, Guangdong Province, China	10	111.441	21.794	4	0.53333 ± 0.180	0.00028 ± 0.00011
32	YN	Yunfu Yunan County, Guangdong Province, China	8	111.586	22.919	1	0.00000	0.00000
33	YX	Yunxiao County, Zhangzhou City, Fujian Province, China	6	117.187	24.061	1	0.00000	0.00000

### DNA extraction, amplification, and sequencing

Total genomic DNA was extracted from *M. officinalis* leaves using the CTAB method (Roy et al., 1992). The *ITS2, rbcL*, *matK* and *trnH-psbA* fragments from each sample were amplified and sequenced. Primers sequences were described previously (Guan et al., 2021). PCR was performed in a 30 μL reaction volume that contained 1 μL of DNA template (20 μg/mL), 2 μL each of primer (10 μmol/L), 15 μL of 2×Hieff PCR Master Mix, and 10 μL of ddH_2_O. PCR involved initial denaturation at 95 °C for 5 min followed by 35 cycles of denaturation at 95 °C for 30 s, annealing at 45 °C for 30 s, extension at 72 °C for 30 s, and final extension at 72 °C for 5 min. PCR products were purified and sequenced using an ABI 3730xL DNA Analyzer (Tianyi Huayu Gene Technology Co., Ltd., Donghu New Technology Development Zone, Wuhan, China). The sequences were deposited in GenBank (PX106935~PX107220 for *ITS2*, PX147468~PX147753 for *rbcL*, PX120918~PX121203 for *matK*, and PX126259XXX~PX126544 for *trnH-psbA*).

### Diversity, haplotype networks, and population structure

Sequences were aligned with MEGA 6 (Tamura et al., 2013) using the CLUSTAL W algorithm, and were edited manually when necessary. Poly-A/T regions and small inversions that generally are highly variable and homoplasic (Kelchner, 2000; Kim and Lee, 2005) were not considered in the analyses. Contiguous insertions/deletions (indels) of more than one base pair were treated as single mutational events (Simmons and Ochoterena, 2000).

The haploid plastid genome does not normally undergo recombination. This uniparental transmission means that the genome is inherited as a unit. The three cpDNA fragments and one nrDNA fragment therefore were concatenated and treated as a single sequence in all analyses. Haplotypes were identified using DNASP 5.10 (Rozas et al., 2003) and relationships between haplotypes were plotted using POPART 1.7 software (Leigh et al., 2015). Indices of haplotypic (h) and nucleotide (π) diversities (Nei, 1987) and inter- and intra-population genetic variation by analysis of molecular variance (AMOVA) were obtained using ARLEQUIN 3.5 (Excoffier and Lischer, 2010). Phylogenetic trees were constructed using Bayesian inference. Bayesian inference and Bayesian posterior probabilities were estimated using MrBayes 3.1.2 (Ronquist and Huelsenbeck, 2003) under the HKY+G model, selected by Modeltest 3.7 (Posada, 2008). Bayesian inference involved 300 million generations, with one tree sampled every 100 generations. Two independent runs were performed using four Markov chains.

The total genetic diversity (HT) of the overall population, the average genetic diversity (HS) within the population, and the coefficient of genetic differentiation between populations (Gst and Nst) were calculated by a permutation test of 1,000 times using PERMUT (Pons and Petit, 1996) software. The probability of a haplotype of a closer relative occurring in a cohort was higher and there was obvious genealogical geographic structure if the Nst value was significantly greater than the Gst value (*P* < 0.05). In contrast, there was no obvious genealogical geographic structure if the Nst value was not significantly greater than the Gst value. The sampled latitude and longitude were converted into a geographic distance matrix using Geographic Distance Matric Generator 1.2.3 software (Ersts, 2025), and then a Mantel test was performed on the geographic and genetic distance matrices of the 33 *M. officinalis* populations using R 4.4.3 (R Core Team, 2025).

### Estimation of divergence time

The dated haplotype phylogenetic trees were estimated by Bayesian inference as implemented in BEAST 1.8.4 (Drummond et al., 2012). Two independent runs were employed, each consisting of 1 × 10^8^ Markov chain Monte Carlo iterations, sampling every 1000 generations under the HKY+G nucleotide substitution model. A lognormal relaxed clock was used, with Yule process as tree prior, and the prior for nucleotide substitution rates utilized a gamma distribution prior with a shape parameter 1.6 and scale parameter 1.6 × 10^−9^ as a prior, assuming an offset value of 1 × 10^−9^ s/s/y. TRACER 1.6 was used to check for convergence of Markov chain Monte Carlo and adequately effective sample sizes (ESS>200) after discarding the first 10% of generations as burn-in. The final joint sample was used to estimate the maximum clade credibility tree using the TREEANNOTATOR software, which is part of the BEAST package, setting 0.5 as limit of posterior probability. Statistical support for the clades was established by assessing the Bayesian posterior probability with node heights summarized to reflect the posterior median. We used *Sansevieria trifasciata* and *Uncaria rhynchophylla* as outgroups (**Supplementary Table S1**). The times of co-differentiation of *M. officinalis* with *S. trifasciata* and *M. officinalis* with the nearest ancestor of *U. rhynchophylla* at 160 Ma Foster and Ho, 2017 and 72 Ma (Ramírez-Barahona, 2017) were used as calibration points.

### Historical dynamics of the population

Neutrality tests using DnaSP 5.10 software (Rozas et al., 2003) were performed to infer possible historical dynamic changes. In addition, ARLEQUIN 3.5 (Excoffier and Lischer, 2010) software was used to analyze the historical dynamics of *M. officinalis* populations using mismatch distribution analysis. This analysis was based on the distribution of base differences between haplotypes, and the fit of expected value and observed value curves were determined. The population size was in a dynamic equilibrium or in a slow decline stage in the long term if the fitted mismatch distribution curves showed double peaks or multiple peaks, whereas the population was recently in an expansion state if a single peak was observed. These two neutral test models were used to assess whether *M. officinalis* populations had expanded in the past. The population was considered to be undergoing bottleneck effects or equilibrium selection when Tajima’s D was significantly greater than 0, but the population was considered to be undergoing expansion or directional selection when Tajima’s D was significantly less than 0. This test is more sensitive and can determine more accurately the historical dynamics of the population. All populations were divided into three subzones when found to be in an expansion period: the continental high-latitude subzone (Guangdong Province, Fujian Province, and eastern Guangxi Zhuang Autonomous Region), the continental low-latitude subzone (southern Guangxi Zhuang Autonomous Region), and the Hainan Island subzone. The more sensitive Bayesian Skyline Plots were utilized to infer changes in the effective population sizes and the timing of the expansion of the populations.

### Ecological niche modeling

Locality data for *M. officinalis* was collected from direct field samples, the China Herbarium Platform (://www.nsii.org.cn/2017/), and the China Digital Herbarium (https://www.cvh.ac.cn/). Records obtained from the databases were verified manually for incongruences, and only those matching species distributions were kept (**Supplementary Table S2**). Only records with global positioning system coordinates and detailed localization were used, which did not compromise the analysis because complete coverage of taxa distribution was still allowed. Explanatory variables included a set of 19 bioclimatic RASTER layers at a 30 arc-second resolution (ca. 1 km^2^ at the equator) from the WorldClim website version 1.4 (Cerasoli et al., 2022). ENM for the current species distribution was performed under three contrasting past climate conditions using a model of maximum entropy (MAXENT 3.3.3) (Phillips et al., 2006), viz. the Last Interglacial (LIG) corresponding to 120,000-140,000 years before present, the LGM corresponding to 21,000 years before present, and the Mid-Holocene (MH) corresponding to 6,000 years before present. The grid layers were cut so as to include the entire geographical distribution for all taxa and were extracted through the RASTER package (Hijmans et al., 2025) implemented in R software. We computed Pearson correlation among all 19 bioclimatic variables using the RASTER package in R (Peterson, 2007). Variables with a correlation coefficient R>0.75 were considered highly correlated. And then from each group of highly correlated variables, we retained the variable that contributed most to the model based on permutation importance (as estimated by MaxEnt in a preliminary run). This approach ensures that we retain the most biologically informative variable from each correlated group while minimizing multicollinearity. The final resulting 10 variables were used in all subsequent ENM analyses to avoid overfitting and improve model interpretability.

The accuracy of model predictions was assessed using the calculated area of the characteristic curve (AUC) values, which ranged from 0~1, with values close to 1 indicating a near-perfect fit. AUC value of approximately 0.5 indicates a random fit whereas a prediction that tends to be systematically incorrect is indicated when the AUC is less than 0.5. The fit generally is considered to be good when the AUC value is between 0.8 and 1. We used this approach to identify areas of high suitability that may harbor taxa during Pleistocene climate change, as well as ecological variables that may explain geographic variation in *M. officinalis*.

## Results

### Genetic diversity, haplotype networks, and genetic structure in *M. officinalis*

The lengths of *ITS2*, *rbcL*, *trnH-psbA*, and *matK* are 438, 644, 319, and 750 bp, respectively. Linking these four regions produced a 2151 bp fragment with 17 polymorphic sites in 33 populations of *M. officinalis*. There were 13 parsimony informative sites among the 17 polymorphic sites, relatively few cpDNA fragment variant sites, and more variant sites in *ITS2* sequences (**Table 1**). Twenty-two haplotypes (H1-H22) were detected (**Table 1**). The DL and DX populations had the highest haplotype and nucleotide diversity among all populations, and 19 populations were free of variation (**Table 1**). The haplotype and nucleotide diversity of *M. officinalis* were 0.569 and 0.00052, respectively. The HS and HT of *M. officinalis* were 0.205 and 0.568, respectively, and the coefficient of genetic differentiation between populations Nst (0.665) > Gst (0.639). These data indicated that these populations had no significant genealogical geographic structure (*P*>0.05). Moreover, AMOVA analysis revealed that there was high genetic differentiation in *M. officinalis* populations (Fst = 0.69263 [*P* < 0.01]), of which 69.26% came from among populations, whereas genetic variation within populations accounted for only 30.74% (**Table 2**). In addition, the gene flow size (Nm) of the *M. officinalis* population was 0.14. The infrequent gene exchange between populations indicated that genetic differentiation of *M. officinalis* mainly existed within populations.

**Table 2:** AMOVA analysis of molecular variation in *Morinda officinalis*.

Source of variation	d.f.	Sum of squares	Variance of components	Percentage of variation
Among populations	32	115.013	0.39489	69.26
Within populations	253	44.337	0.17524	30.74
Total	285	159.350	0.57014	
Fixation Index	F_ST_:0.69263**			

**P < 0.01.

Mantel tests based on the correlation between genetic and geographic distances showed that the genetic distance among *M. officinalis* populations weak positively correlated with the geographic distance (*P* = 0.01, r=0.3632) (**Supplementary Figure S1**).

### Phylogeny analysis reveals population expansion in *M. officinalis*

**Figure 2:**
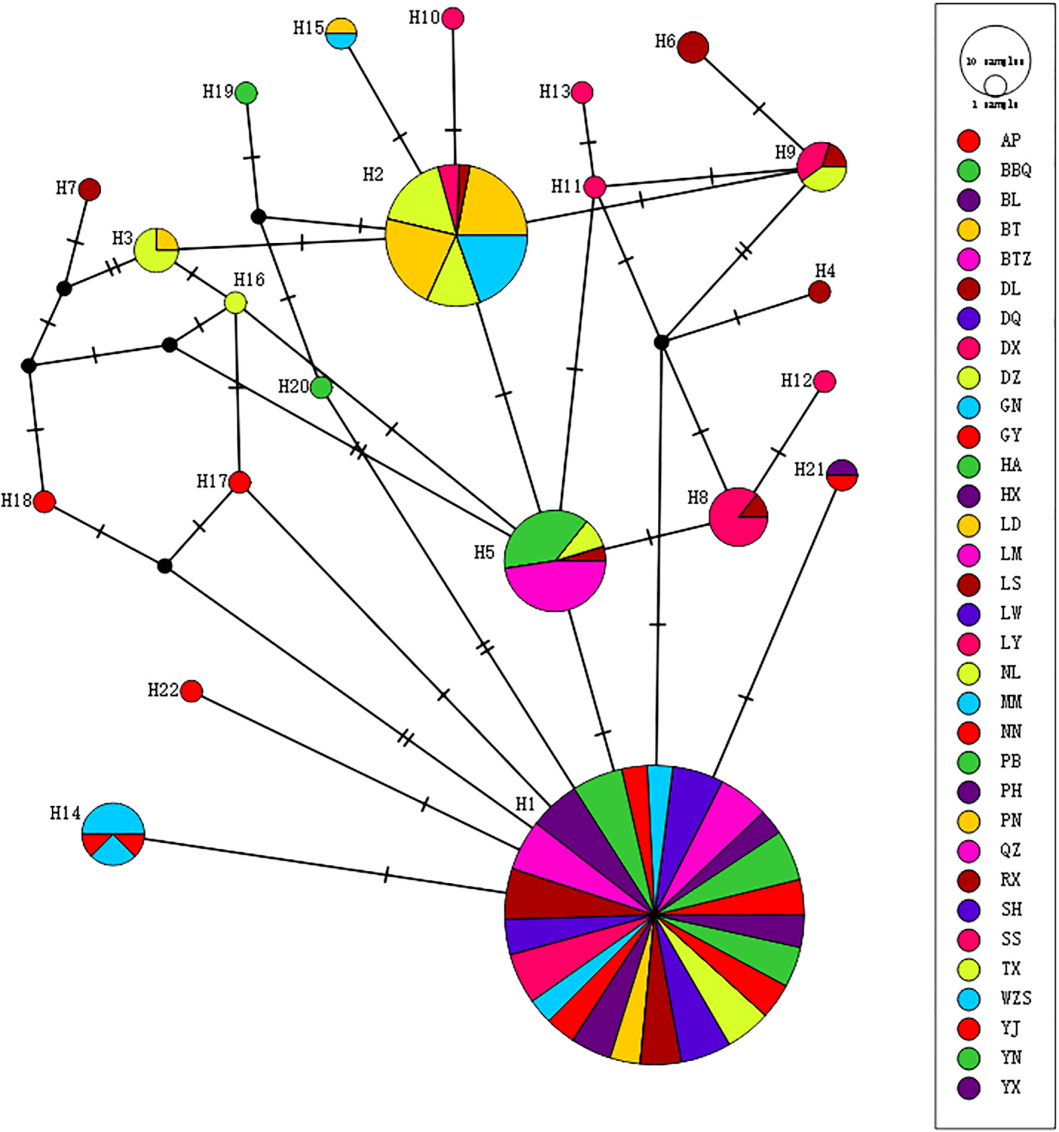
Haplotype network diagram of *Morinda officinalis*. Pie chart sizes represent haplotype frequencies for each population. The abbreviation of this group name is shown in the **Table 1**.

Analysis of the phylogenetic relationships among haplotypes revealed that H1 and H2 were the most common and widespread in the sampled populations of *M. officinalis* (**Figure 2**). H1 occupied the center of the network and was distributed in Guangdong Province and Fujian Province, as well as in eastern Guangxi Zhuang Autonomous Region. H2 was distributed in the Shiwan mountains and in the Liuwan mountains from the islands to southern Guangxi Zhuang Autonomous Region.

**Figure 3:**
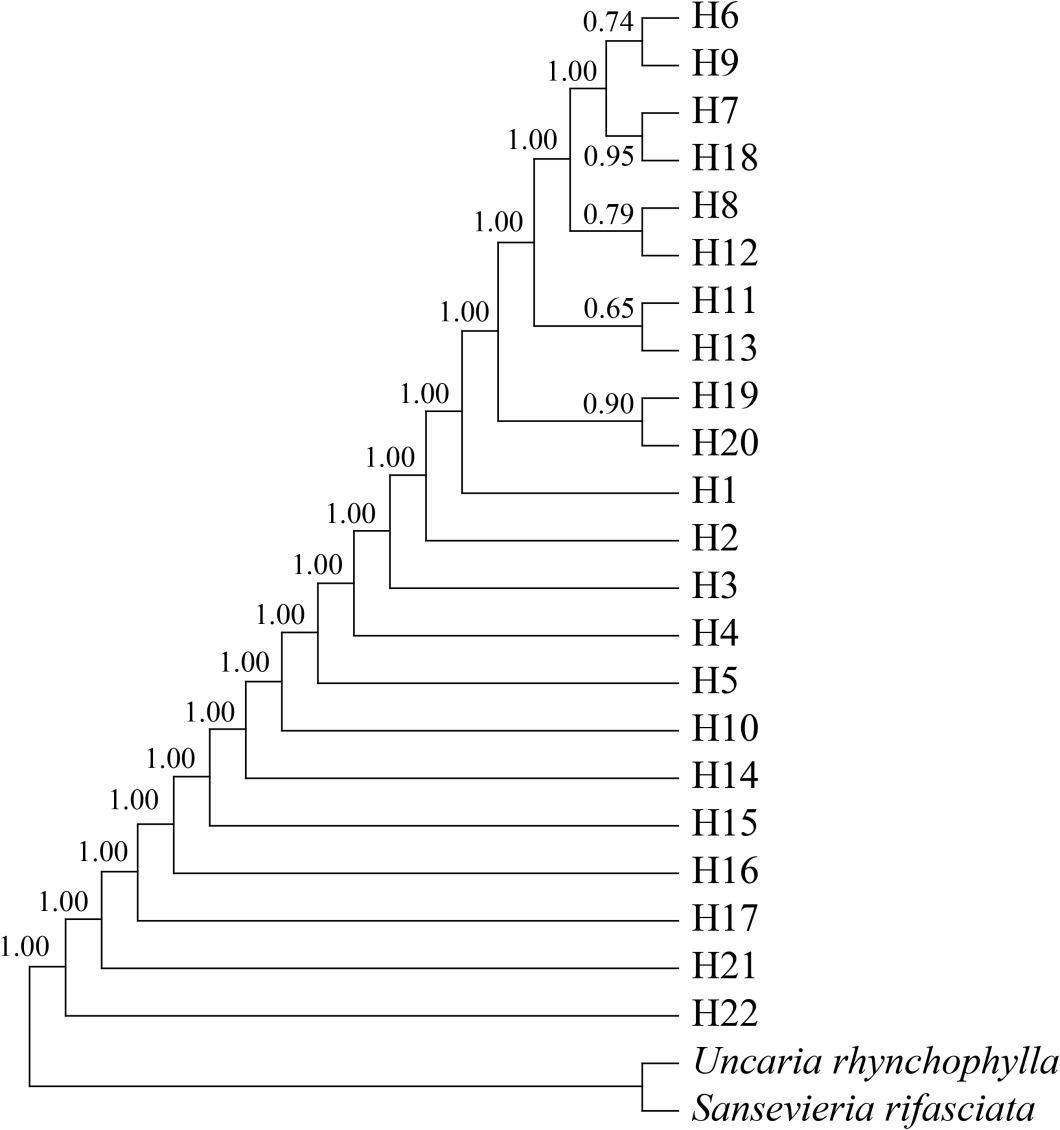
Bayesian haplotypes tree of *Morinda officinalis*. H6-H9, H11-H13, and H18-H20 are from south-central Guangxi Zhuang Autonomous Region, whereas other haplotypes are from Provinces of Guangdong and Fujian, eastern Guangxi Zhuang Autonomous Region, and Hainan Island. The value displayed above the node is the support rate. The abbreviation of this group name is shown in the **Table 1**.

Bayesian interference analysis showed a support node of bootstrap support >0.5, with the 22 haplotypes of *M. officinalis* clustered into a large branch (**Figure 3**). Although overall differentiation is not obvious, there is support for the five small branches composed of 10 haplotypes from south-central Guangxi (H6-H9, H11-H13, and H18-H20), which indicate that genetic diversity of *M. officinalis* in this region is higher and with close relatives, thus showing genealogical structure. In contrast, the populations in Guangdong Province, Fujian Province, eastern Guangxi Zhuang Autonomous Region, and Hainan Island are almost undifferentiated (**Figure 3**).

### Historic dynamics of the *M. officinalis* population

**Figure 4:**
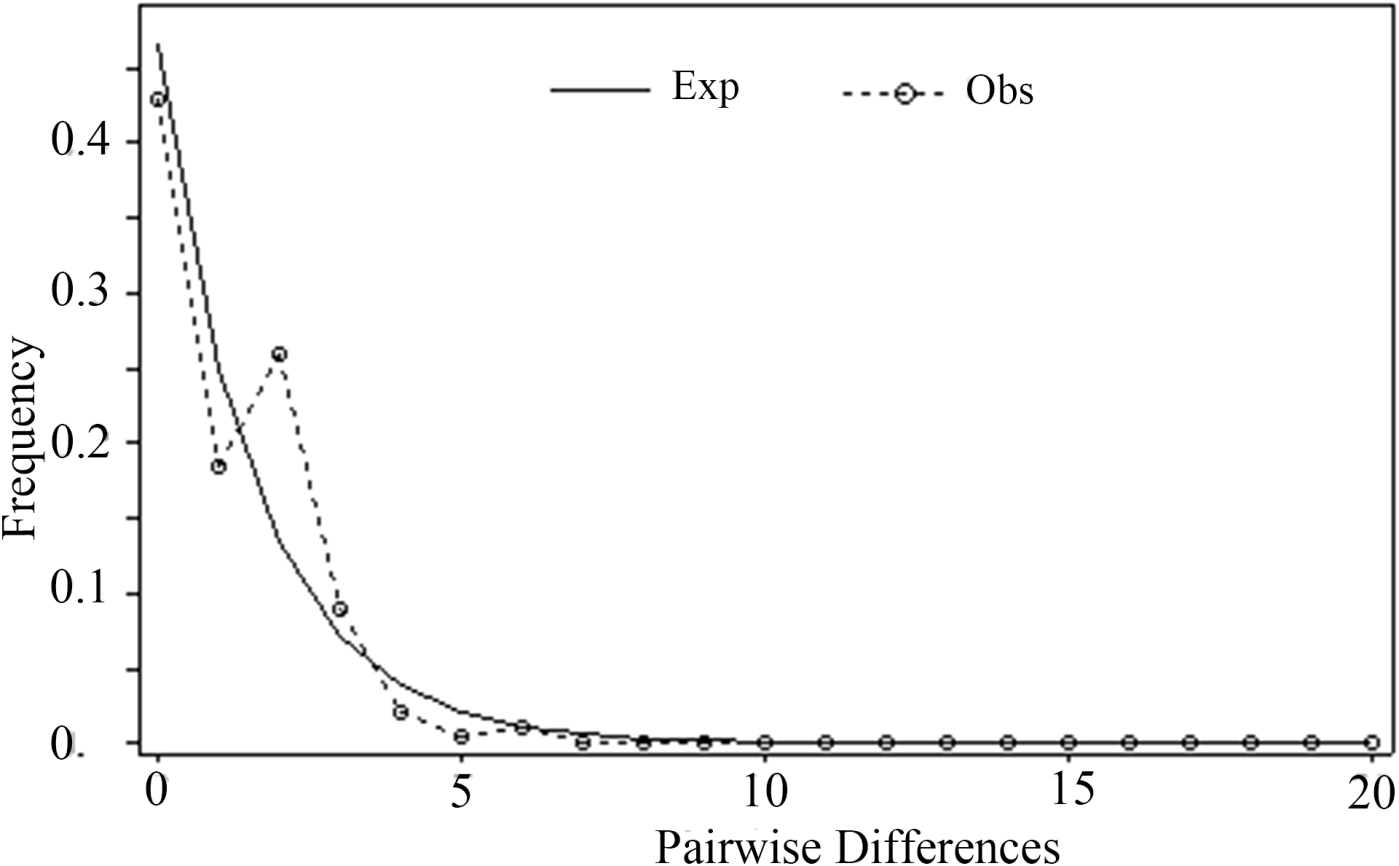
Analysis of mismatch distribution in the *Morinda officinalis* populations.

**Figure 5:**
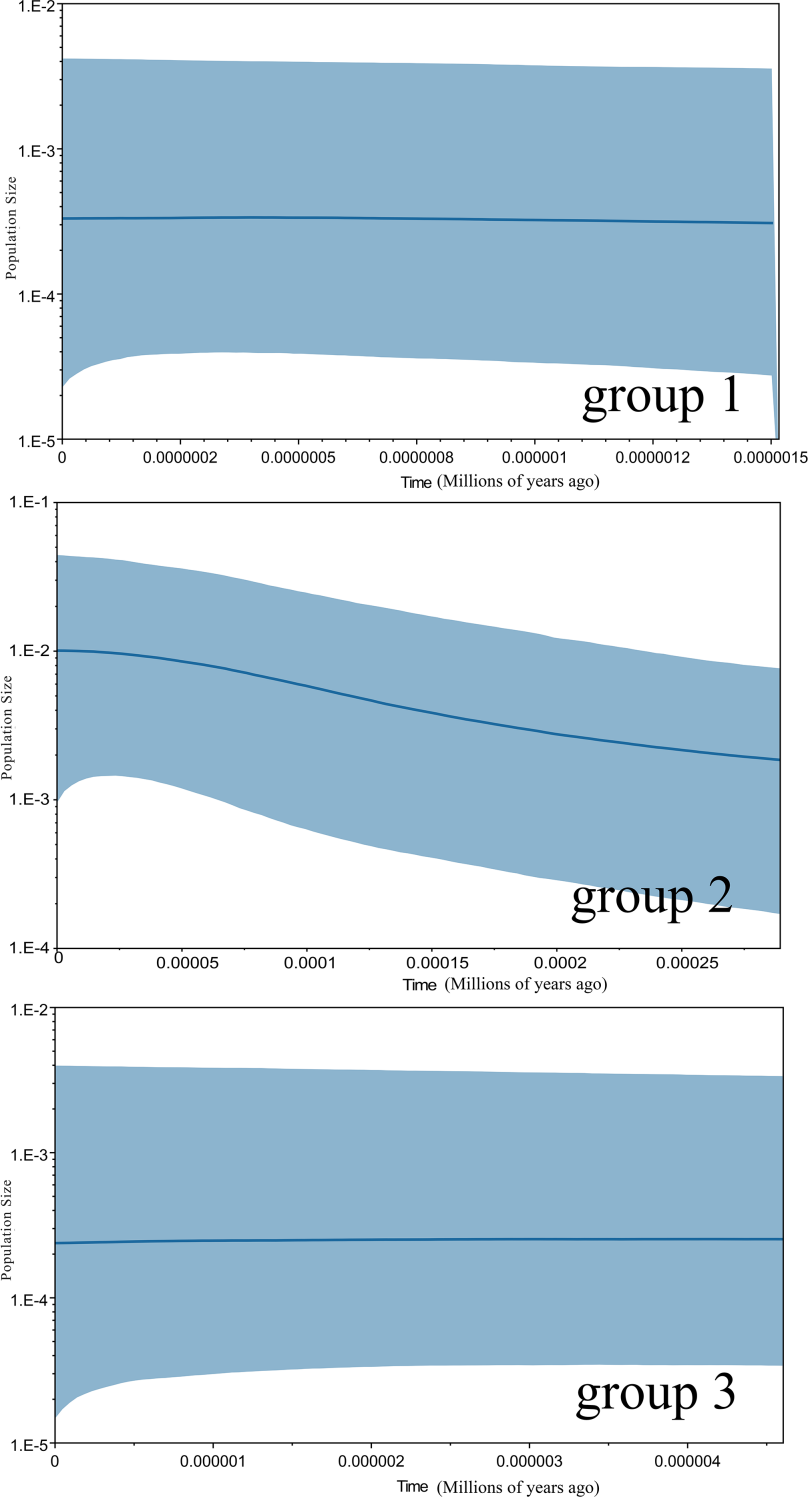
Extended Bayesian skyline plots showing the demographic trends in groups 1, group 2 and group3 of *Morinda officinalis*. The x-axis is in units of million years ago and the y-axis represents the estimated population size on a log scale (Net/106). The central line shows the median estimate of effective population size, while dashed lines represent the 95% credibility limits. Group 1, populations from Provinces of Guangdong Fujian, and the eastern Guangxi Zhuang Autonomous Region; Group 2, populations from southern Guangxi Zhuang Autonomous Region; Group 3, populations from Hainan Island in China.

The frequency distribution of paired nucleotide differences between individual haplotypes was calculated to test the hypothesis of population expansion in *M. officinalis*. The distribution of mismatches was clearly unimodal (**Figure 4**), which suggests that the *M. officinalis* population underwent expansion. A neutrality test revealed that Tajima’s D value was non-significant (-1.71275, *P*>0.05), Fu and Li’s D* value showed significant negative correlation (-2.42023, *P* < 0.05), and Fu and Li’s F* value also was correlated negatively (-2.52864, *P* < 0.05), which further indicate population expansion in the southern Guangxi Zhuang Autonomous Region. According to the Bayesian skyline plot (**Figure 5**), the effective population size of *M. officinalis* increased rapidly in the southern Guangxi Zhuang Autonomous Region with swift population expansion events. In contrast, bottleneck effects or equilibrium selection were observed in *M. officinalis* populations in Guangdong Province, Fujian Province, the eastern Guangxi Zhuang Autonomous Region, and Hainan Island.

### Divergence time estimations of *M. officinalis*

**Figure 6:**
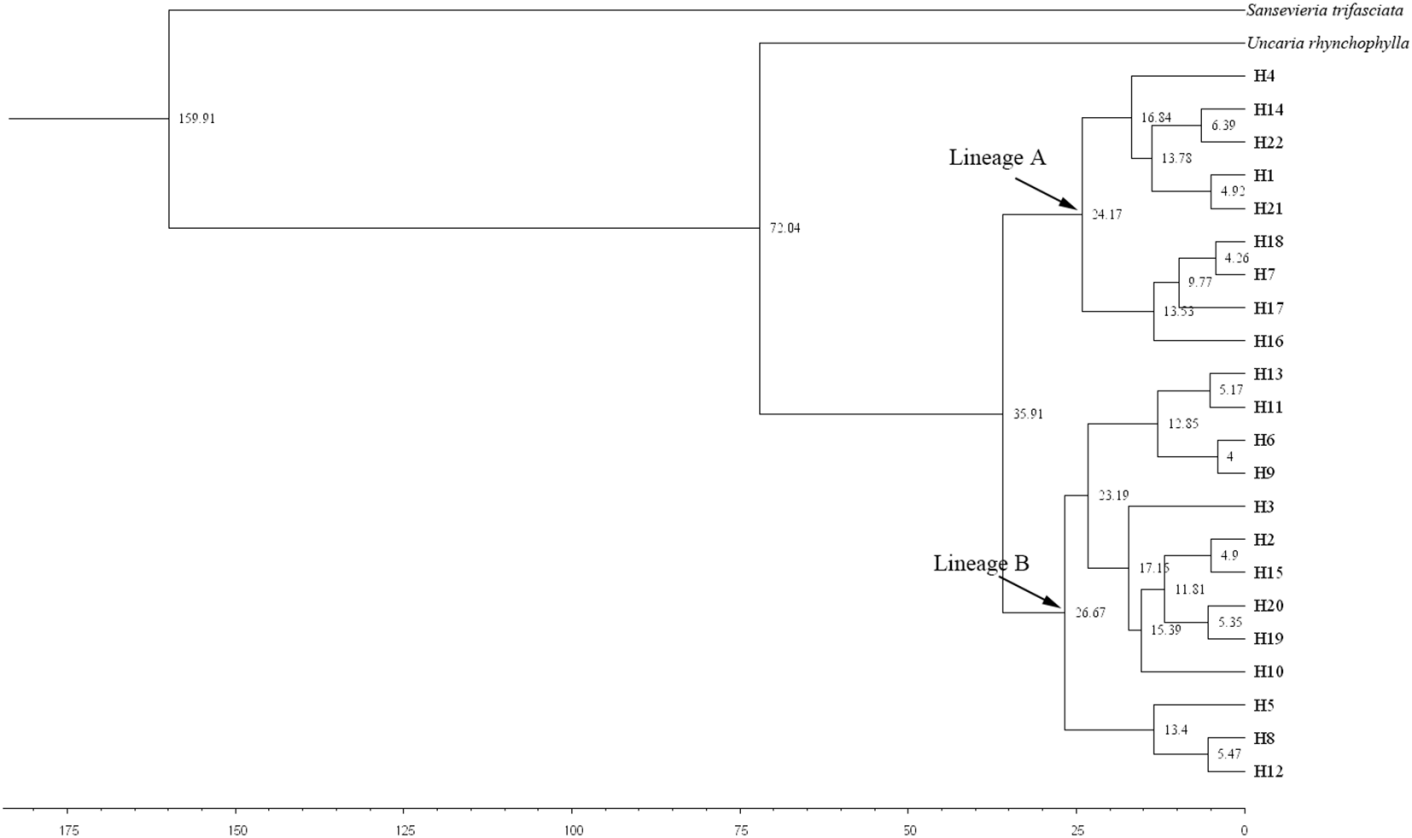
Differentiation time (Mya) of *Morinda officinalis* haplotypes estimated by BEAST. The value displayed above the node is the support rate. The abbreviation of this group name is shown in the **Table 1**.

The divergence time of *M. officinalis* from the same family of plants, i.e., *U. hynchophylla* (Hooker), was approximately 72.04 Mya (**Figure 6**). The appearance time of the common ancestor of *M. officinalis* is 35.91 Mya, with subsequent division into Lineages A and B with divergence times of 24.17 and 26.67 Mya, respectively. Lineage A continued to divide into two subclades, with divergence times of 16.84 and 13.53 Mya, whereas Lineage B diversified into three subclades, with divergence times of 12.85, 13.4 and 17.15 Mya (**Figure 6**).

### Ecological niche modeling of *M. officinalis* populations

The potential distribution of ecological niche models of *M. officinalis* was shown in **Figure 1**. The mean AUC value was higher than 0.97 in all analyses, indicating a high level of predictive accuracy. Comparisons between models of *M. officinalis* populations under different climatic conditions showed significant differences in the present, Holocene (~6 kya), LGM (~21 kya) and LIG (~70 kya) fitness zones, which indicate that the distribution of this species has experienced fluctuating increments (**Figure 1**). The current core distribution area of *M. officinalis* essentially is the same as the existing distribution of the species. The fitness area (reaching moderate fitness) is distributed mainly in suitable areas (reaching moderate suitability) in Guangdong Province, Guangxi Zhuang Autonomous Region, Fujian Province, Hainan Province, and Taiwan Province, with some additional distribution in Yunnan Province. Under LIG climatic conditions, the suitable zones for *M. officinalis* are mainly in the Kunlun Mountains and Bayan Kela Mountains on the Tibetan Plateau and in the Hengduan Mountains in Yunnan Province, with some distribution on Hainan and Taiwan islands. The suitable area during this time was 9.1329×10^5^ km^2^, which is appreciably less than the current suitable area (13.88×10^5^ km^2^). Under the climatic conditions of LIG, the suitable area (reaching moderately suitable area) is essentially similar (10^5^ km^2^). Under LGM climatic conditions, *M. officinalis* has a suitable distribution area of 23.41×10^5^ km^2^, which is an increase in area compared with that of the LIG. The distribution range in this case is on the Tibetan Plateau where survival of the species is threatened by snow and ice cover. The *M. officinalis* population in the western part of the Tibetan Plateau retreated to nearby refuges due to these threats, whereas the population in the Bayan Kra and Hengduan Mountains expanded southeastward to southeastern China and formed refuges. Under MH climatic conditions, the suitable area in the Tibetan Plateau was reduced drastically due to uplift of the Plateau and the impacts of global warming. The total area of distribution is only 10.62×10^5^ km^2^, which accounts for only 45.36% of the LGM. The optimal distribution area is located mainly in the vicinity of the Kunlun Mountain Range and southern Guangxi, with small distribution areas in southern Guangdong, Hainan, and Taiwan. Compared to the MH, the current distribution area of *M. officinalis* has increased to 13.88×10^5^ km^2^, with the Kunlun Mountain Range as the main refuge. The optimal distribution area is near the Kunlun Mountains moving southeastward to Guangdong Province, Guangxi Zhuang Autonomous Region, Fujian Province, Hainan Province, and Taiwan Province (**Supplementary Table S3**).

## Discussion

### Genetic diversity, haplotype networks, and population structure of *M. officinalis*

The genetic diversity of plant species is influenced to varying degrees by geographical distribution, breeding methods, and population size. This study analyzed the combined sequences of cpDNA and ITS in *M. officinalis*, with HT = 0.568 and HS = 0.205, which are lower than the average chloroplast genetic diversity of 170 other plant species (HT = 0.670) (Petit et al., 2005). Moreover, genetic diversity in *M. officinalis* is less than in *Gynostemma pentaphyllum* (cpDNA, HT = 0.912) (Su, 2020) and *Siraitia grosvenorii* (cpDNA, h=0.735; CHS, h=0.914; EDL2, h=0.834) (Jie, 2019), which have similar geographical distributions. However, compared with plants in the same family, genetic diversity is higher than in *Gardenia jasminoides* (nrDNA, HT = 0.246) (Liu et al., 2022), which indicates that *M. officinalis* has a longer species evolutionary history compared to closely related plants.

The population of *M. officinalis* has a lineage geographic structure, but this structure is not significant statistically. However, there is a significant correlation between the genetic and geographical distances of *M. officinalis*. The inter-population variation (69.26%) is more than the intra-population variation (30.74%) according to AMOVA analysis, which indicates a high level of genetic differentiation among different populations. The haplotype network diagram of *M. officinalis* shows that haplotype H1 is the source of expansion and has the highest distribution frequency. This haplotype exists in populations in Guangdong Province, Fujian Province, and eastern Guangxi Zhuang Autonomous Region, followed by haplotype H2, which is distributed in southern Guangxi Zhuang Autonomous Region and Hainan Island. There is no coexistence of H1 and H2 in a population. The differences between these haplotypes comprise G-to-A mutations at both 1810 bp and 1962 bp of the *ITS2* sequence in the tandem fragment. The *ITS2* region sequence is relatively well-conserved and may reflect genetic stability within the species. However, there also is a modest degree of stable intraspecific variation, which suggests that there is lineage differentiation between *M. officinalis* samples from Hainan Island, southern Guangxi Zhuang Autonomous Region, Guangdong Province, Fujian Province, and eastern and central Guangxi Zhuang Autonomous Region.

*M. officinalis* flowers usually are monoecious and have the ability to self-pollinate as well as the potential to cross-pollinate within the *M. officinalis* population, which is reflected in the greater gene flow (Nm=0.14) compared to self-pollinating plants (Nm=0.065), but less than of cross pollinating plants (Nm=5.38). These observations are consistent with gene flow in the grasses *Stipa krilov* and *Stipa capillata* (Peng et al., 2015; Zheng et al., 2009). Evolutionary history, life history characteristics, reproductive systems, and habitat distribution range exert significant impact on population genetic structure (Zhang et al., 2014; Gogi et al., 2018). The high Fst value (0.69263) indicates substantial genetic differentiation among populations, consistent with limited gene flow (Nm = 0.14) and long-term isolation. However, the non-significant difference between Nst and Gst (P > 0.05) suggests a lack of strong phylogeographic structure, likely due to the patchy distribution of karst habitats and the absence of a continuous geographic cline. These patterns reflect the combined effects of genetic drift in isolated populations and the species limited dispersal ability, rather than isolation-by-distance.

High genetic differentiation among populations of *M. officinalis* suggest that the species has an evolutionary history of approximately 50.39 Ma, although cpDNA has a slower evolutionary rate (Petit et al., 2005), which generates less genetic diversity than from nrDNA(*ITS2*) sequences. However, due to the accumulation of considerable genetic variation over a long period of evolutionary history, genetic differentiation of this species at the cpDNA and nrDNA(*ITS2*) sequence levels is relatively high. High genetic differentiation among populations of *M. officinalis* also may reflect that the species is monoecious and that close encounters with neighboring plants may lead to substantial pollen flow between different individuals within the population. *M. officinalis* is distributed mainly in the hills of Lingnan, usually growing in high mountains and valleys. The Qiongzhou Strait acts as a natural barrier that effectively hinders the spread of seeds and pollen, which results in severe habitat fragmentation. Although the fruit of *M. officinalis* may be ingested by fruit-eating animals, including birds, monkeys, and bats, and spread over long distances through excretion, the transmission efficiency is low. Effective gene exchange between populations has not been achieved, which further exacerbates the significant genetic differentiation among *M. officinalis* populations.

### Phylogenetic inference and patterns of *M. officinalis* population distribution

The distribution pattern of *M. officinalis* revealed that the genetic diversity of populations in Guangdong Province, Fujian Province, and the eastern part of the Guangxi Zhuang Autonomous Region was low, whereas genetic diversity conversely was high in the southern part of the Guangxi Zhuang Autonomous Region. Lower temperatures or ice caps covered the original habitats of *M. officinalis* at the onset of the ice age, which forced the species to migrate to lower elevations or latitudes or to warmer refuges. Species spread from refuges to other suitable habitats as ice caps melt, which may lead to a founder effect that results in northern populations with reduced genetic diversity (Hewitt, 2000, 2004). In addition, Hainan Island, which represents the lowest-latitude range of *M. officinalis*, exhibits notably low genetic diversity. This pattern can be attributed to a combination of historical biogeographic processes and recent anthropogenic impacts. Geologically, volcanic activity raised sea levels approximately 2.0-2.5 Mya (Yeh, 1986; Ke, 1983). Subsequent sea level changes during the Pleistocene led to multiple separations of the island from mainland China. The Middle Pleistocene land bridge was formed three times at 0.6-0.8, 0.42-0.48, and 0.13-0.3 Mya (Jin et al., 1982). Hainan Island was also connected to the mainland during the Last Glacial Period (0.015-0.025 Mya) (Shi et al., 2002), but separated at 0.0071-0.01 Mya (Ke, 1983). This recurring geographic isolation significantly limited gene flow, promoting genetic drift and reducing diversity. The presence of early-diverged haplotypes such as H5 and H15 suggests that *M. officinalis* likely colonized Hainan Island prior to the Early Pleistocene and was subsequently isolated by the formation of the Qiongzhou Strait. In addition to these historical factors, the species is constrained by its specialization on karst limestone habitats, which are naturally fragmented on the Hainan Island. Furthermore, the low genetic diversity observed in Hainan Island populations is consistent with patterns seen in other species on the island that have been severely impacted by anthropogenic activities (e.g. *Angelica sinensis*, Zhang et al., 2014). While the primary drivers of the initial genetic structure in *M. officinalis* are likely historical isolation and genetic drift, the fragile insular habitat of Hainan is highly susceptible to human disturbance. Anthropogenic activities, including overharvesting, habitat fragmentation, and the introduction of exotic species, have further reduced genetic diversity in already vulnerable populations. This is especially evident in Hainan Island, where small population sizes and narrow distribution ranges amplify the effects of genetic drift and inbreeding. Human-mediated habitat degradation likely accelerated the loss of rare haplotypes and reduced overall genetic variability. Therefore, the current genetic patterns on Hainan are likely the result of a combination of deep historical processes and recent anthropogenic pressures.

### Historical dynamics of the *M. officinalis* population

It has been proposed that southern China was not covered by an ice sheet during the last glacial period, but that instead the climate was 4-6 °C colder than currently with a marked drying out that caused species in this region to undergo complex climatic and vegetative changes throughout the glacial cycle (Hewitt, 2000; Shi, 2002; Yan et al., 2012; Qiu et al., 2011; Yan et al., 2012; Tian et al., 2015; Xu et al., 2015; Denk and Grimm, 2009; Dong, 2023; Gong et al., 2016; Tzedakis et al., 2002; Meng et al., 2015; Herzschuh et al., 2010; Hughes and Eastwood, 2006; Antonelli et al., 2009). It has been demonstrated that subtropical plants located in the area conform to the expansion-contraction model (Chaves et al., 2011; Li et al., 2012). *M. officinalis* belongs to the broad group of heat- and moisture-loving plants that mainly are distributed in the tropics and subtropics, which is in line with the expansion-contraction model. The complex topography and landscape of southern China, as well as the existence of many east-west trending mountain ranges, including the Wuyi and Nanling Mountain ranges, make the climate of the Ice Age less impactful and the hills of southeastern China may be a potential refuge during this time. The Nanling Mountains have been reported as a refuge for many plants during the Ice Age (Favre et al., 2015; Ferriol et al., 2004; Gao et al., 2007). Here, we observed that the highest haplotype diversity in *M. officinalis* was in the DL population in southern Guangxi Zhuang Autonomous Region (Hd=0.9643), followed by the DX population (Hd=0.9286). Nucleotide diversity was also highest in the DL population (0.0015), followed by the DX population (0.0001). High and low levels of nucleotide diversity tend to reflect the dynamic history of populations, and areas with elevated nucleotide diversity and haplotype polymorphisms may be ice age refuges for species (Jin et al., 2003). Nucleotide diversity was also high in most of the *M. officinalis* populations near southern Guangxi Zhuang Autonomous Region, which is characterized by mountainous terrain, commonly known as the Shiwan Mountains, which have a separating effect on the populations. These mountain ranges serve as geographical barriers and protect species by reducing the impact of strong climatic fluctuations during the ice age (Posada and Crandall, 2001). *M. officinalis* haplotype diversity gradually increased from the northeast to the southeast of its distribution range and reached a maximum in Fangchenggang City, Guangxi Zhuang Autonomous Region, and a fragmentary increase in the vicinity of Guangning County, Guangdong Province and Yangjiang City, Guangdong Province. Combining the haplotype diversity and nucleotide diversity of the populations, we hypothesize that the Liuwan and Shiwan mountains in southern Guangxi Zhuang Autonomous Region, the Dinghu Mountains in Zhaoqing, Guangdong Province, and the mountains near the Goohuang Roach in Yangjiang, Guangdong Province, may have been the principal ice age refuges of *M. officinalis* during the Quaternary Ice Age.

Our results challenge the classical paradigm of ‘southward contraction–northward expansion’ that has been widely applied to temperate species in East Asia (e.g., *Ginkgo biloba*; Gong et al., 2008). Instead, the phylogeographic and ENM evidence supports a southward expansion of *M. officinalis* during the LGM, likely driven by increased aridity in northern latitudes and the availability of suitable microrefugia in complex topographic regions such as the Nanling and Shiwan Mountains. This pattern aligns with recent studies of other subtropical evergreens (e.g., *Cyclobalanopsis glauca*; Zhang et al., 2022) and underscores the importance of ecological specificity and regional heterogeneity in shaping biogeographic responses to climate fluctuations. Thus, our study contributes to a refined model for East Asian flora, in which subtropical species may exhibit distinct range dynamics that deviate from temperate-centric paradigms.

The uplift of the Tibetan Plateau is one of the most prominent recent global geological events. With an average elevation of more than 4,500 m and covering an area of 2.3 million km^2^ (Bittkau and Comes, 2005), the area currently is the highest and largest plateau in the world and possesses exceptional geological features. Paleogeographic events, including the upliftment of mountain ranges and alteration of drainage systems, are other drivers that affect present-day patterns of biogenetic diversity and have led to habitat fragmentation and the formation of barriers to gene flow. These factors promote genetic differentiation and even species formation in plants, with a particular impact on plant differentiation in southwestern China (Hughes and Eastwood, 2006; Antonelli et al., 2009; Chaves et al., 2011). The divergence of the two major clades of *M. officinalis* at 35.91 Mya, followed by the separation of Lineages A and B at 26.67 Mya and 24.17 Mya, shows a notable correspondence with a major period of Tibetan Plateau uplift and the establishment of the Asian monsoon system (35–20 Mya; Favre et al., 2015). Lineage A separated further into two subclades with divergence times of 16.84 and 13.53 Mya, and Lineage B branched into three subclades with divergence times of 12.85, 13.4, and 17.15 Mya. At this time in the Late Miocene or Early Pliocene, which corresponds to a period of Tibetan Plateau high-altitude mountain range uplift and Central Asian aridification (20–10 Mya; Favre et al., 2015), further uplift of the Plateau and Himalayas during the Pliocene (2.59-5.30 Mya) posed a significant challenge to plant survival (Ferriol et al., 2004). Haplotypes within the *M. officinalis* sub-branch diverged rapidly during this time. Historical orogenic movements, climate change, and environmental heterogeneity all have major impacts on the genetic structure of species populations and haplotype differentiation (Gao et al., 2007; Jin et al., 2003; Posada and Crandall, 2001). The timing of intraspecific divergence in *M. officinalis* coincided with paleogeographic events on the Tibetan Plateau, and thus population dispersal and evolution were influenced by the uplift of the Tibetan Plateau, possibly since the Late Eocene. The results of ENM indicated that the suitable area for *M. officinalis* was distributed mainly on the Tibetan Plateau during the LIG, and that this area began to migrate to the southeastern part of China during the LGM. The climate during the LIG was warmer than the current climate (Gao et al., 2007; Hewitt, 2000; Shi, 2002; Qiu et al., 2011). Therefore, the suitable distribution area for *M. officinalis* was still centered on the Tibetan Plateau during the last interglacial period. However, the climate in southern China was colder by 4-6°C during the LGM than that today even though this region was not covered by an ice cap (Hewitt, 2000; Shi, 2002; Qiu et al., 2011; Yan et al., 2012). The suitable distribution area of *M. officinalis* was mainly in the Tibetan Plateau during the LGM when a large-scale southeastern migration of *M. officinalis* occurred. The ENM results under past climate changes offer insights into the species’ vulnerability to future warming. The observed reliance of *M. officinalis* on stable, humid subtropical niches suggests that current core areas such as Guangdong Province and Guangxi Zhuang Autonomous Region may experience range contractions due to increased temperature extremes and seasonal droughts. Conversely, higher elevation regions or currently marginal habitats may become increasingly suitable. However, the species’ limited dispersal capacity and high habitat specificity could severely constrain natural range shifts. These implications highlight the urgency of integrating climate resilience into conservation planning, including *ex situ* preservation, assisted migration, and the protection of potential future refugia.

Although the ENM result provides valuable insights into the climatic suitability for *M. officinalis*, it does not incorporate soil properties (e.g., calcium availability in karst regions) or biotic interactions (e.g., pollinator or disperser availability), which are known to influence its distribution. However, our ENM results suggest that current core distribution areas (e.g., Guangdong Province and Guangxi Zhuang Autonomous Region) may face range contractions under future climate warming due to increased thermal stress and hydrological changes. Conservation efforts should prioritize genetic reserve establishment in stable refugial areas and consider assisted migration to newly suitable regions. Future studies could enhance model accuracy by integrating high-resolution soil data and proxies for biotic interactions where available. Nonetheless, our models still captured major range shifts consistent with phylogeographic patterns, suggesting that climate has been a primary driver of historical distribution changes.

Crustal movement or sea level rise lead to the separation of islands from the mainland, which renders islands a natural laboratory for studying the effects of isolation on species formation (Bittkau and Comes, 2005). Thus, the separation of Hainan Island from the mainland by the Qiongzhou Strait blocked the exchange of genes between island and inland populations. We found four haplotypes (H2, H3, H5, and H15) in the five populations of *M. officinalis* on Hainan Island. Haplotypes H2 and H5 also are present in southern Guangxi Zhuang Autonomous Region, whereas H3 and H15 are endemic to Hainan Island. Hainan Island has a fragile habitat and field surveys demonstrated that the small plant population and narrow distribution range make the plants in this location more vulnerable to extinction than those on land. Plants on the island also are influenced by frequent human activity and the introduction of a large number of exotic plants and animals. The endemic haplotypes on the island have become endangered, which may lead to a decline in island biodiversity or even species extinction (Francisco-Ortega et al., 2000). Therefore, in exploring the genealogical history of any taxon, the spatial and temporal context of these processes must be considered, and anthropogenic factors should be included and viewed from a dialectical perspective. The emergence of humans and the increasing human population have become the dominant factors in environmental change, and alterations in natural landscapes have been caused mainly by human intervention (Triantis and Mylonas, 2009). The genetic diversity of species is vulnerable to human activities.

## Conclusions

We applied for the first time an integrated approach to explore the effects of past climatic events and recent geological features on the phylogeographic patterns of the tropical and subtropical species *M. officinalis* in China. The study revealed correlations between geological events and patterns of genetic variation in the species. *M. officinalis* clade divergence coincided with times of geological events, most notably movements in the Tibetan Plateau region. Thus, the occurrence of major geological events disrupted *M. officinalis* habitats and formed geographic barriers, which restricted gene flow due to geographic isolation. In addition, *M. officinalis* exhibits weak dispersal ability that compounds the effects of geographic distance as a barrier to gene exchange in the species. Differences in microenvironments in different geographic regions after differentiation give rise to local adaptations that manifest as morphological differences. Our study similarly explored the relationship between climatic events and patterns of genetic variation. The southern region of China is characterized during Quaternary climatic conditions by the presence of numerous high mountain ranges whose complex structures provide suitable microhabitats to escape from the unfavorable climatic conditions of the Pleistocene Ice Age fluctuations. The *M. officinalis* population became stabilized in southern China because the high and low altitude regions provided sufficient buffers for species survival in the face of climate change. Future studies incorporating populations from Taiwan are essential to obtain a comprehensive phylogeographic perspective.

In summary, geological events and Quaternary climate fluctuations are the main parameters that affected the pattern of genetic variation of *M. officinalis* in southern China. Crustal movements or sea level rises and anthropogenic factors also exert influences on the pattern of genetic variation in southern China, which provides important insights into the mechanism of genetic variation in *M. officinalis* and other plant species in the region.

## Data Availability

The datasets presented in this study can be found in online repositories. The names of the repository/repositories and accession number(s) can be found in the article/**Supplementary Material**.
