# Supplementary material for: Phylogeography and ecological niche modeling suggest southward expansion of Morinda officinalis How in China
Source: Front Plant Sci. 2025 Nov 21;16:1643733. doi: 10.3389/fpls.2025.1643733 (PMC12678398; doi:10.3389/fpls.2025.1643733)

Table S1 GenBank accession number of Outgroups sequences

| Sequence | GenBank accession number | |
| --- | --- | --- |
|  | *Sansevieria trifasciata* | *Uncaria rhynchophylla* |
| rbcL | ON934287.1 | KM057029.1 |
| matK | KX783780.1 | KM057054.1 |
| trnH-psbA | FN675812.1 | OK420698.1 |
| ITS2 | OP410917.1 | AJ346900.1 |

Table S2 Species records obtained from the Herbarium Platform and the China Digital Herbarium

| Number | Longitude | Latitude |
| --- | --- | --- |
| 1 | 110.85 | 26.43 |
| 2 | 106.75 | 25.43 |
| 3 | 110.235565 | 24.175587 |
| 4 | 110.585686 | 25.60168 |
| 5 | 107.957979 | 22.097768 |
| 6 | 117.31 | 24.7 |
| 7 | 109.83 | 19.03 |
| 8 | 110.311897 | 26.355276 |
| 9 | 110.050532 | 25.255089 |
| 10 | 111.566656 | 24.403562 |
| 11 | 110.0400066 | 25.742153 |
| 12 | 112.0440245 | 22.9293756 |
| 13 | 108.6537981 | 21.9816823 |
| 14 | 109.7003424 | 18.6263091 |
| 15 | 109.8544195 | 24.0454286 |
| 16 | 111.0316515 | 22.4465422 |
| 17 | 113.4150624 | 24.1860449 |
| 18 | 104.691 | 38.0733 |
| 19 | 111.441 | 21.794 |
| 20 | 107.78 | 21.75 |
| 21 | 109.531 | 22.079 |
| 22 | 110.841 | 22.981 |
| 23 | 110.335 | 22.958 |
| 24 | 108.135 | 21.87 |
| 25 | 107.995 | 21.607 |
| 26 | 116.886 | 24.476 |
| 27 | 117.167 | 24.132 |
| 28 | 110.674 | 23.711 |
| 29 | 111.064 | 23.163 |
| 30 | 111.586 | 22.919 |
| 31 | 112.287 | 23.171 |
| 32 | 112.387 | 23.537 |
| 33 | 111.385 | 21.842 |
| 34 | 114.313 | 23.597 |
| 35 | 114.176 | 23.239 |
| 36 | 115.806 | 23.192 |
| 37 | 111.622 | 23.999 |
| 38 | 111.938 | 23.318 |
| 39 | 107.63 | 21.785 |
| 40 | 108.383 | 22.856 |
| 41 | 117.291 | 24.909 |
| 42 | 117.747 | 24.567 |
| 43 | 117.187 | 24.061 |
| 44 | 117.413 | 24.713 |
| 45 | 108.755 | 18.502 |
| 46 | 109.283 | 19.896 |
| 47 | 109.583 | 18.718 |
| 48 | 109.657 | 19.086 |
| 49 | 109.688 | 18.914 |
| 50 | 112.528 | 23.335 |
| 51 | 117.636 | 25.006 |
| 52 | 117.2139 | 24.9115 |
| 53 | 119.2861 | 28.305 |
| 54 | 115.8581 | 24.9111 |
| 55 | 111.2833 | 25.5052 |
| 56 | 111.2575 | 25.4986 |
| 57 | 109.6497 | 25.0286 |
| 58 | 107.8628 | 21.8394 |
| 59 | 105.7758 | 23.2822 |
| 60 | 111.5539 | 24.6201 |
| 61 | 107.0133 | 21.8539 |
| 62 | 107.5325 | 21.7114 |
| 63 | 104.81 | 22.95 |
| 64 | 101.4167 | 21.6833 |

Table S3 Predicted area of potential suitable distribution areas for *Morinda officinalis* in different periods

| Period | Prediction area（×10^5^ km^2^） | | | | |
| --- | --- | --- | --- | --- | --- |
|  | Lowly  suitable area | Sub-suitable area | Highly  suitable area | The most  suitable area | Total suitable area |
| Current | 82.1239 | 8.0665 | 2.8441 | 2.9655 | 13.8760 |
| MH | 85.0571 | 6.0100 | 2.5765 | 2.0326 | 10.6191 |
| LGM | 72.6213 | 15.3848 | 7.8022 | 0.2222 | 23.4095 |
| LIG | 86.8235 | 7.1528 | 1.4594 | 0.5208 | 9.1329 |

Figure S1 Mantel test of geographical distance and genetic distance among *M. officinalis* populations.


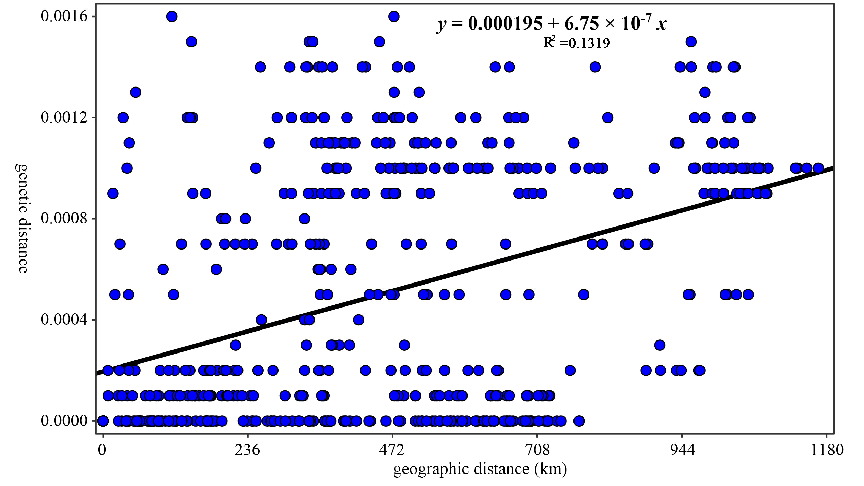

Supplement: Supplementary file 1 [file Table1.docx]
